# Downregulated mRNA Expression of ZNF385B Is an Independent Predictor of Breast Cancer

**DOI:** 10.1155/2021/4301802

**Published:** 2021-02-03

**Authors:** Ning Yan, Cong Liu, Fang Tian, Ling Wang, Yimin Wang, Zhaoying Yang, Yan Jiao, Miao He

**Affiliations:** ^1^Department of Breast Surgery, China-Japan Union Hospital of Jilin University, Changchun, Jilin 130033, China; ^2^Department of Obstetrics and Gynecology, The Second Hospital of Jilin University, Changchun, Jilin 130022, China; ^3^Scientific Research Center, China-Japan Union Hospital of Jilin University, Changchun, Jilin 130033, China; ^4^Department of Hepatobiliary and Pancreatic Surgery, The First Hospital of Jilin University, Changchun, Jilin 130021, China; ^5^Department of Anesthesia, The Second Hospital of Jilin University, Changchun, Jilin 130022, China

## Abstract

**Background:**

ZNF385B, a zinc finger protein, has been known as a potential biomarker in some neurological and hematological studies recently. Although numerous studies have demonstrated the potential function of zinc finger proteins in tumor progression, the effects of ZNF385B in breast cancer (BC) are less studied.

**Methods:**

The Oncomine database and “ESurv” tool were used to explore the differential expression of ZNF385B in pan-cancer. Furthermore, data of patients with BC were downloaded from The Cancer Genome Atlas (TCGA). The receiver operating characteristic (ROC) curve of ZNF385B expression was established to explore the diagnostic value of ZNF385B and to obtain the cut-off value of high or low ZNF385B expression in BC. The chi-square test as well as Fisher exact test was used for identification of the relationships between clinical features and ZNF385B expression. Furthermore, the effects of ZNF385B on BC patients' survival were evaluated by the Kaplan-Meier and Cox regression. Data from the Gene Expression Omnibus (GEO) database were employed to validate the results of TCGA. Protein expression of ZNF385B in BC patient specimens was detected by immunohistochemistry (IHC) staining.

**Results:**

ZNF385B expression was downregulated in most types of cancer including BC. Low ZNF385B expression was related with survival status, overall survival (OS), and recurrence of BC. ZNF385B had modest diagnostic value, which is indicated by the area under the ROC curve (AUC = 0.671). Patients with lower ZNF385B expression had shorter OS and RFS (relapse-free survival). It had been demonstrated that low ZNF385B expression represented independent prognostic value for OS and RFS by multivariate survival analysis. The similar results were verified by datasets from the GEO database as well. The protein expression of ZNF385B was decreased in patients' samples compared with adjacent tissues by IHC.

**Conclusions:**

Low ZNF385B expression was an independent predictor for worse prognosis of BC patients.

## 1. Introduction

Breast cancer (BC) is the most common female carcinoma [1] and has become a concerned health issue globally in recent years. In fact, BC has been reported as the first cause of cancer death among women across the world now [2]. With the groundbreaking medical advances during the past decades, people have realized that BC is a complicated disease with various stages and subtypes [3]. Approaches to dealing with BC are moving in a more effective and accurate direction. However, detection and prognosis of BC are still challenging. Thus, identifying biomarkers with clinical significance for the diagnosis and prognosis of BC is strongly demanded.

Malignant proliferation is always shadowed by the essential alterations in genome changes. ZNF385B, also called ZNF533 [4], belongs to a member of the zinc finger gene (ZNF) family. ZNF385B played a vital role in gene expression, working as encoding transcription factors [5]. Henriette found that trimethylation of lysine 9 (H3me3K9), which is associated with silenced chromatin, was highly enriched in ZNFs. ZNFs, as promoters of transcription factor, were also combined with the corepressor KAP1. Briefly, Krüppel-associated box domain zinc finger (KRAB-ZNF) family members were involved in a self-regulatory loop and led to H3K9 trimethylation and transcriptional inhibition which indicated that this mRNA may be a transcription suppressor [6]. In recent studies, it has already been reported in several systemic disease, including Burkitt' s lymphoma, cardiac arrest, nonsyndromic orofacial clefts, and obesity [4, 7–9]. However, the potential effects of ZNF385B expression in BC have not been defined. In the present research, we found differential expression of ZNF385B in multiple types of cancer including BC compared to normal tissues using the Oncomine database [10] and significant prognostic value of ZNF385B in BC based on a web-based tool “ESurv” [11]. In addition, a research based on the prognostic value of 8-gene for early BC found that ZNF385B may show some value in the prognostic scoring of early BC, but it remains to be further validated [12]. However, the diagnostic and prognostic value of ZNF385B for BC and the associations between ZNF385B and clinicopathological parameters in BC patients have not been reported. We discussed ZNF385B mRNA expression between BC patients and healthy human beings by analyzing TCGA (The Cancer Genome Atlas) and GEO (Gene Expression Omnibus) databases, and its expression was verified by immunohistochemistry (IHC) staining using patients' samples. To identify the diagnostic value of ZNF385B, patients were split into high or low group based on ZNF385B expression. Besides, we also explored the connections between ZNF385B expression and clinicopathologic factors as well as survival times including overall survival (OS) and relapse-free survival (RFS) of BC patients. Our results showed that ZNF385B might serve as a potential diagnostic and prognostic biomarker of BC.

## 2. Methods

### 2.1. Pan-Cancer Analysis of ZNF385B Based on Oncomine Database and “ESurv” Tool

The Oncomine database (http://www.oncomine.com) was adopted to evaluate the difference of ZNF385B mRNA expression levels between tumor and normal tissues in various tumor types. The analysis conditions were set as follows: *p* < 0.0001, fold change > 2, gene rank (top 10%), and data type (all). The web-based tool “ESurv” (https://www.giantonline.org) was used to perform the survival analysis of ZNF385B in pan-cancer systemically as well.

### 2.2. Data Mining and Analysis in TCGA and GEO Databases

The level 3 expression data of BC was obtained from TCGA database (https://cancergenome.nih.gov/), and the data underwent log_2_ (*x* + 1) transformation for RSEM normalized counts. Clinical data of BC patients were also obtained from TCGA database. The processing and analysis of all data were performed with R software [13]. To validate the results in TCGA database, the GSE21422 [14] and GSE20711 [15] datasets were obtained from the GEO database (http://www.ncbi.nlm.nih.gov/geo/). Correlations between ZNF385B expression and pathological variables such as ER, HER-2, grade, and subtype were explored. The effects of ZNF385B on OS and RFS were assessed as well.

### 2.3. Immunochemistry (IHC) Staining

A total of 10 paired tumor and adjacent tissues of patients with BC were collected. All patients received surgery at the China-Japan Union Hospital of Jilin University and were histologically diagnosed by 2 independent pathologists based on WHO criteria. IHC staining was performed to identify the expression level of ZNF385B in BC patients' tissues. Briefly, 3 *μ*m thick sections were deparaffinized, rehydrated, and submerged into EDTA for antigen retrieval. All sections were next dealt with hydrogen, heated, incubated in bovine serum albumin, and then followed by incubation with ZNF358B antibody (Abcam, Cambridge, MA, USA) at 4°C overnight. Normal goat serum served as negative control. The sections were washed and subsequently cultivated with secondary antibody and incubated with horseradish peroxidase-streptavidin complex (Invitrogen, CA, USA). Each section was immersed in 3-amino-9-ethyl carbazole followed by counterstaining with Mayer's hematoxylin, dehydrating, and mounting. Nuclear staining was considered positive.

### 2.4. Statistical Analysis

The expression of ZNF385B in patients was evaluated through box plots. The value of ZNF385B in diagnosis was assessed with AUC (area under the curve) by establishing the ROC (receiver operating characteristic) curve through the pROC package. According to the threshold value confirmed by the ROC curve, patients were classified into low or high ZNF385B expression group. The chi-square test as well as Fisher exact test was used for analysis of the associations between clinicopathologic features and ZNF385B expression in BC. Using the survival package in R, OS and RFS were compared via Kaplan-Meier analysis between the high and low ZNF385B expression groups, and the *p* value was calculated via log-rank test. Potential prognostic factors were screened via univariate Cox analysis followed by further survival evaluation using multivariate Cox analysis. *p* value < 0.05 signified statistical significance.

## 3. Results

### 3.1. Pan-Cancer Analysis of ZNF385B mRNA Expression

Firstly, the Oncomine database was used to assess the differential expression of ZNF385B mRNA levels in various cancers, and low ZNF385B expression was found in multiple types of cancer, such as BC, brain malignant tumor, lung cancer, kidney cancer, and liver cancer (Supplementary Figure 1A). Then, single gene analysis was performed in pan-cancer about ZNF385B using the web-based tool “ESurv” [11], and it was found that aberrant expression of ZNF385B was related to poor prognosis of BC, renal cancer, liver cancer, and brain cancer (Supplementary Figures 1B–1E).

### 3.2. Characteristics of the Study Population

Clinical data of 1104 BC patients and 114 normal controls were obtained from TCGA database. All data are shown in Table 1, including age, gender, menopause status, histological type, T classification, N classification, M classification, TNM stage, molecular subtype, ER, PR, HER-2, margin status, vital status, radiation therapy, neoadjuvant treatment, targeted molecular therapy, sample type, OS, RFS and ZNF385B expression of BC. And all the patients were assigned to different groups based on these characters.

### 3.3. ZNF385B Expression in BC

**Figure 1:**
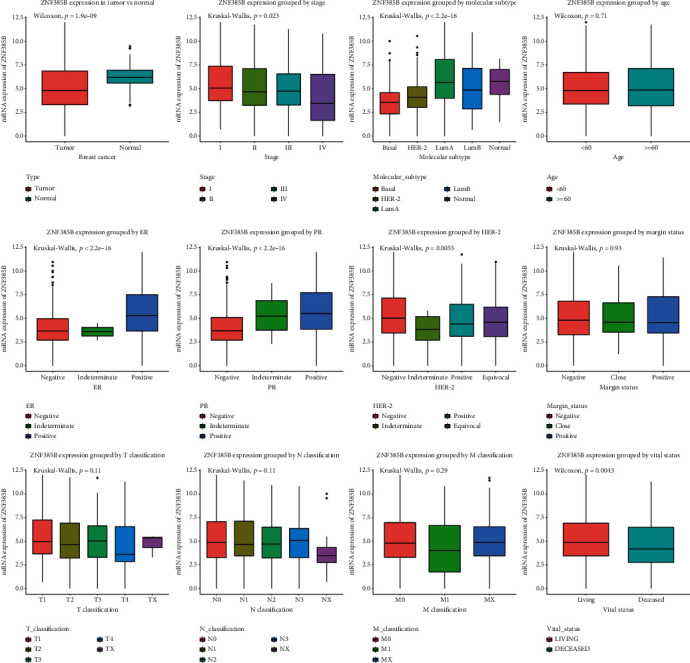
ZNF385B expression in breast tumor. The expression of ZNF385B is significantly lower in tumor than normal tissue. Low ZNF385B expression closely related with TNM stage, molecular subtype, ER, PR, HER-2 and vital status.

ZNF385B expression status between BC and normal tissues is shown in Figure 1, which demonstrated downregulation of ZNF385B expression in BC (*p* < 0.0001). Furthermore, differences in ZNF385B expression were also observed in molecular subtype (*p* < 0.0001), ER (*p* < 0.0001), PR (*p* < 0.0001), TNM stage (*p* = 0.023), HER-2 (*p* = 0.0056), and vital status (*p* = 0.0043). To better determine the role of ZNF385B expression in predicting prognosis in BC patients, the ROC curves were performed to determine the optimal cut-off values for high and low ZNF385B expression. The cut-off value for high and low ZNF385B expression was 3.439 for TCGA data and 4.403 for GEO dataset (Supplementary Figure 2).

### 3.4. Diagnostic Value of ZNF385B Expression in BC

**Figure 2:**
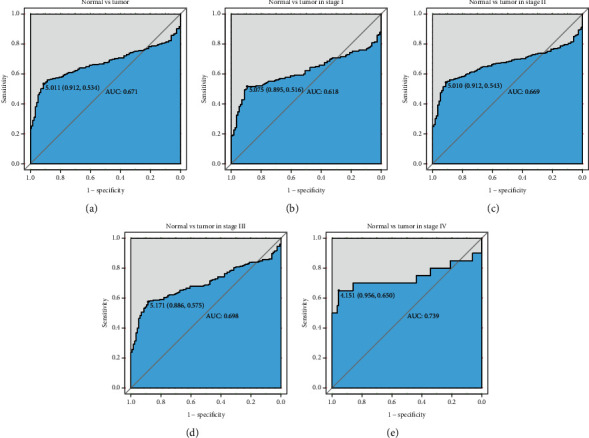
The diagnostic value of ZNF385B expression. (a) ROC curve for ZNF385B expression in breast cancer and normal tissue. (b–e) Different stages of breast cancer also showed certain diagnostic value.

The ROC curve was plotted to evaluate the diagnostic value of ZNF385B based on the expression data from healthy individuals and BC patients (Figure 2(a)). It showed modest diagnostic value concluded from AUC with 0.671. We can observe that ZNF385B expression in different stages of BC also showed certain diagnostic value via subgroup analysis with AUC value of 0.618 for stage I, 0.669 for stage II, 0.698 for stage III, and 0.739 for stage IV (Figures 2(b)–2(e)).

### 3.5. Connection between Clinicopathologic Features and ZNF385B Expression in BC

The association between clinicopathologic parameters and ZNF385B expression of BC cases from TCGA database is shown in Table 2. The following features had significant correlation with ZNF385B expression including histological type, molecular subtype, ER, PR, vital status, OS, RFS (all *p* < 0.001), and TNM stage (*p* = 0.039).

### 3.6. Low ZNF385B Expression Showed Independent Prognostic Value of BC Patients for OS

**Figure 3:**
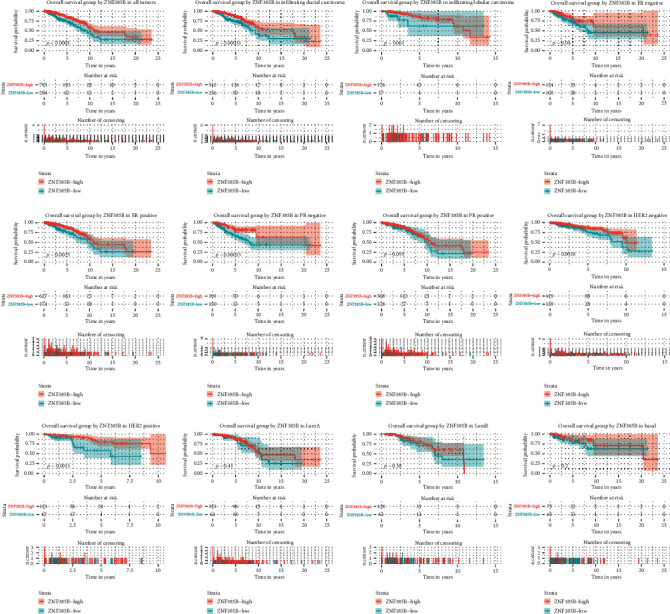
Kaplan-Meier curves of OS in breast cancer. Kaplan-Meier curves for OS in all tumors, different histological types (infiltrating ductal carcinoma and infiltrating lobular carcinoma), ER, PR, HER-2, and molecular subtype (LumA, LumB, and basal).

The impact of ZNF385B expression on OS was evaluated through the Kaplan-Meier curve which indicated that low ZNF385B expression related with worse OS (*p* < 0.0001) (Figure 3). Furthermore, low ZNF385B expression significantly affected the OS in infiltrating ductal carcinoma (*p* = 0.00031), ER-negative BC (*p* = 0.04), ER-positive BC (*p* = 0.0025), PR-negative BC (*p* = 0.00015), HER-2-negative BC (*p* = 0.0038), and HER-2-positive BC (*p* = 0.0013) through subgroup analysis. Univariate and multivariate analyses were carried out to evaluate the prognostic value of clinicopathologic parameters. As shown in Table 3, the univariate analysis showed that age, menopause status, T classification, N classification, M classification, TNM stage, margin status, and ZNF385B expression were all meaningful prognostic factors for OS. Furthermore, multivariate analysis indicated that ZNF385B expression was an independent predictor for OS of BC patients (HR = 3.04, 95% CI: 1.894-4.877, *p* < 0.001). Besides, age, N classification, M classification, and margin status also showed independent prognostic value for OS.

### 3.7. Low ZNF385B Expression Showed Independent Prognostic Value of BC Patients for RFS

**Figure 4:**
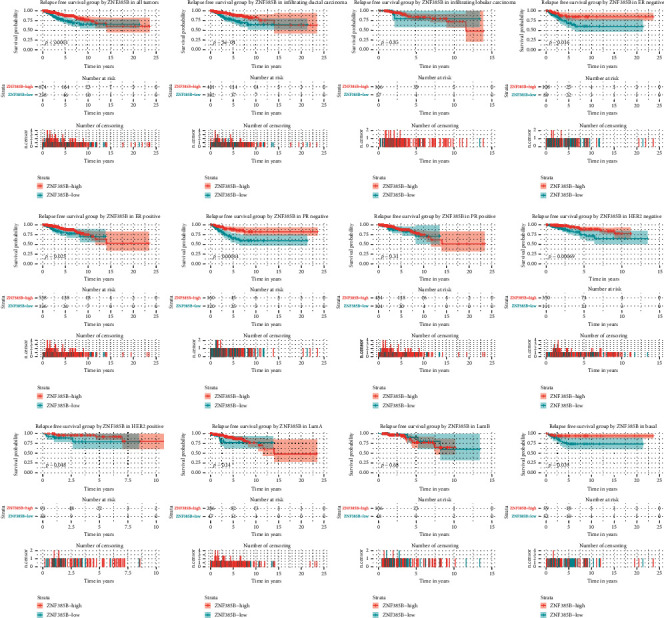
Kaplan-Meier curves of RFS in breast cancer. Kaplan-Meier curves for RFS in all tumors, different histological types (infiltrating ductal carcinoma and infiltrating lobular carcinoma), ER, PR, HER-2, and molecular subtype (LumA, LumB, and basal).

The Kaplan-Meier curves indicated that low ZNF385B expression had relation to poorer RFS (*p* < 0.0001) (Figure 4). In addition, low ZNF385B expression significantly affected the RFS in infiltrating ductal carcinoma (*p* < 0.0001), ER-negative BC (*p* = 0.016), ER-positive BC (*p* = 0.025), PR-negative BC (*p* = 0.00044), HER-2-negative BC (*p* = 0.00069), HER-2-positive BC (*p* = 0.048), and basal-like BC (*p* = 0.039) through subgroup analysis. Univariate and multivariate analyses were used to confirm the prognostic value of clinicopathologic parameters. As shown in Table 4, the univariate analysis showed that ER, PR, T classification, N classification, M classification, TNM stage, margin status, and ZNF385B expression were all meaningful prognostic factors for RFS. Furthermore, multivariate analysis manifested that ZNF385B was an independent predictor for RFS of BC patients (HR = 2.609, 95% CI: 1.531-4.449, *p* < 0.001). Besides, N classification and margin status also showed independent prognostic value for RFS.

### 3.8. ZNF385B Expression and Pathological Characteristics of BC Patients in GEO Databases

**Figure 5:**
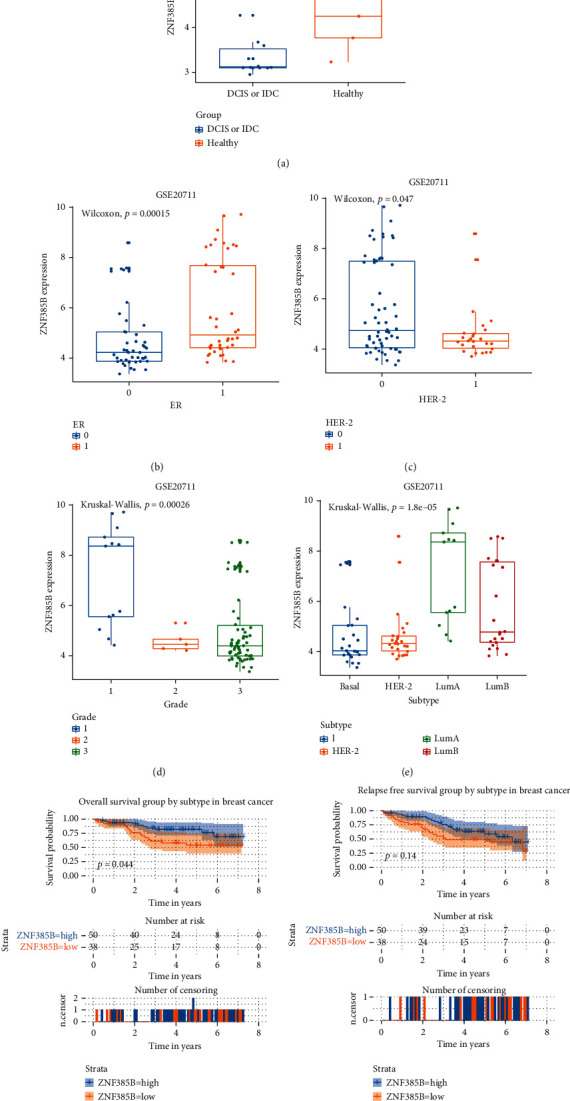
Association between mRNA expression levels of ZNF385B and pathological parameters of breast cancer. The parameters included ER, HER-2, grade, and subtype. Low ZNF385B expression related with short OS. The validation data from GEO database were analyzed. *p* < 0.05 was considered as statistically significant.

We obtained GEO datasets to further investigate the role of ZNF385B in BC (Figure 5). The results were broadly consistent with our findings above. The validation via the microarray GSE21422 demonstrated that ZNF385B expression levels in tumor tissues, which included invasive ductal carcinoma (ICD) and ductal carcinoma in situ (DCIS), were lower than those in healthy samples (*p* = 0.03; Figure 5(a)). Differential levels of ZNF385B expression were presented in the following variables (validated by microarray GSE20711): ER status (*p* = 0.00015), HER-2 status (*p* = 0.047), grade (*p* = 0.00026), and subtype (*p* < 0.0001) (Figures 5(b)–5(e)). Besides, ZNF385B expression showed differences in survival analysis as well. Patients with lower ZNF385B expression level had shorter OS (*p* = 0.044; Figure 5(f)).

### 3.9. Low ZNF385B Expression in BC Patient Tissue Samples

**Figure 6:**
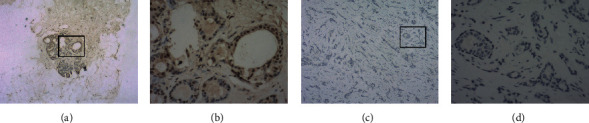
Immunohistochemistry staining for ZNF385B. The expression of ZNF385B in breast cancer cell was decreased. (a, b) IHC staining of paratumor breast tissues ((a) ×40, (b) ×400); (c, d) IHC staining of breast cancer tissues ((c) ×40, (d) ×400).

IHC staining was performed to verify the expression of ZNF385B in BC patients' tissue samples. ZNF385B presented high expression in paratumor tissues when detected by IHC staining (Figures 6(a) and 6(b)), while low expression in BC tissues (Figures 6(c) and 6(d)). All paratumor breast tissues presented positive nuclear staining (*n* = 10), and most of the tumor tissues presented negative nuclear staining (*n* = 8) and others with weak positive staining (*n* = 2).

## 4. Discussion

Our present research was the first to find the difference of ZNF385B expression between BC patients and healthy individuals. To our knowledge, our research was the first to link ZNF385B to BC. It was found that aberrant expression of ZNF385B was associated with BC significantly based on a web-based tool “ESurv” [11] and Oncomine database [10]. We then further validated the relationship between ZNF385B expression and diagnosis as well as prognosis of BC using data from TCGA and GEO databases. We found that low ZNF385B expression related with poor survival status and recurrence of patients with BC. Besides, ZNF385B expression represented modest diagnostic value in BC using ROC analysis. The Kaplan-Meier curves for OS and RFS revealed that lower ZNF385B expression had relation to worse prognosis in BC patients. We obtained the similar results verified by datasets from the GEO database as well. Besides, the low expression of ZNF385B was verified in BC patients' samples using IHC. It has been showed that ZNF385B could work as an independent biomarker for BC prognosis through univariate and multivariate Cox analysis.

In human genome, zinc finger proteins have been known as one of the largest transcription factor family [16]. The functions were diverse, such as RNA packaging, DNA recognition, transcriptional activation, protein folding assembly, regulation of apoptosis, and lipid binding [17]. Zinc finger proteins showed diversity in multiple biological processes as a result of a complex combination and function of zinc finger motifs, which included development, differentiation, metabolism, and autophagy. In recent years, more and more reports have revealed the potential function of zinc finger proteins in tumor progression; however, the mechanisms were different, even in different types of cancer and the same cancer under different types of stress.

ZNF385B, whose encoded protein sequences with 4 matrin-type zinc fingers are highly conserved, is located in the AUTS5 region (2q31.2-q31.3) [18]. ZNF385B was considered to be a potential transcriptional repressor, while its target genes have not been identified [6]. It has been reported that ZNF385B had three isoforms. Isoform- (IF-) 1, containing four ZF domains, was the longest transcript variant. And IF-2/3 was shorter which contained three ZF domains. ZNF385B IF-1 could influence p53 and mediate apoptosis through upregulating PERP and FAS/CD95 and then activated caspase-8 and caspase-3. We supposed that transcriptional inhibition decreased neoplasia and metastasis of tumor cells in some way. Thus, tumor growth and metastasis could be promoted when the transcriptional repression of ZNF385B reduced and mRNA levels raised and resulted in shorter OS.

ZNF385B was expressed in adult brain tissue widely; besides, it related with the development of the lip and palate as well [19]. In the field of oncology, Elgaaen BV et al. first reported that in serous ovarian carcinomas, ZNF385B was lowly expressed and correlated with survival [5]. In addition, ZNF385B is expressed only in Burkitt lymphoma cells, but hardly in diffuse large B-cell lymphoma [4]. In the current genome-wide copy number variation analysis, containing 2319 individuals with BRCA1 pathogenic variants, it was reported that ZNF385B might relate with BC via qPCR and/or nanosorting analysis [20]. However, the position of ZNF385B in diagnosis and prognosis of BC has not been defined. Our present study showed that ZNF385B was expressed at low level in BC. ROC analysis has provided evidence for ZNF385B as a potential diagnostic biomarker of BC. We found a feasible relationship between ZNF385B and survival in BC by connecting ZNF385B expression with survival status as well. Subgroup analyses showed that low ZNF385B expression significantly affected the OS and RFS in many BC subtypes such as infiltrating ductal carcinoma, ER-negative BC, ER-positive BC, PR-negative BC, HER-2-negative BC, and HER-2-positive BC, and its low expression showed worse prognosis in those patients. As we all know, molecular subtype has become the standard for guiding treatment options of BC patients [21], and different therapies such as chemotherapy and endocrine therapy have been developed to better treat patients with BC based on it [22]. Proper molecular typing and accurate prognosis are essential for cancer treatment [23]. Therefore, it is of great significance to research new prognosis-related genes for prognosis assessment and treatment guidance for BC and its different subtypes. In our study, low expression of ZNF385B has shown important prognostic value in different subtypes, which might be used to evaluate the risk and prognosis of subgroups and guide beneficial individual treatment options for patients with high risk of death or worse prognosis [24].Currently, surgery has been regarded as an important measure for malignant tumor. However, the possibility of recurrence especially in basal-like BC could adversely influence the outcome of patients. In this report, the connections between ZNF385B expression and recurrence in different subtypes of BC were explored. Low expression of ZNF385B affected RFS in patients who suffered basal-like BC, while it less affected luminal A or luminal B subtype, which indicated the specific prognostic value of ZNF38B.

Overall, our study provided evidence that ZNF385B showed important value in diagnosis and prognosis of patients with BC. However, further experimental confirmation is required to verify the conclusion.

## 5. Conclusion

Our research indicated that ZNF385B was downregulated in BC. Low ZNF385B expression might be related to clinical progression and work as a potential biomarker in diagnosis and prognosis of BC patients.

## Figures and Tables

**Table 1:** Characteristics of the study population.

Characteristics	Numbers of cases (%)
Age	
NA	2 (0.18)
<60	589 (53.35)
≥60	513 (46.47)
Gender	
NA	2 (0.18)
Female	1090 (98.73)
Male	12 (1.09)
Histological type	
NA	3 (0.27)
Infiltrating ductal carcinoma	790 (71.56)
Infiltrating lobular carcinoma	204 (18.48)
Other	107 (9.69)
Molecular subtype	
NA	255 (23.1)
Basal	142 (12.86)
HER-2	67 (6.07)
Luminal A	422 (38.22)
Luminal B	194 (17.57)
Normal	24 (2.17)
ER	
NA	50 (4.53)
Indeterminate	2 (0.18)
Negative	239 (21.65)
Positive	813 (73.64)
PR	
NA	51 (4.62)
Indeterminate	4 (0.36)
Negative	345 (31.25)
Positive	704 (63.77)
HER-2	
NA	183 (16.58)
Equivocal	180 (16.3)
Indeterminate	12 (1.09)
Negative	565 (51.18)
Positive	164 (14.86)
Menopause status	
NA	93 (8.42)
Indeterminate	34 (3.08)
Peri	40 (3.62)
Post	706 (63.95)
Pre	231 (20.92)
T classification	
NA	2 (0.18)
T1	281 (25.45)
T2	640 (57.97)
T3	138 (12.5)
T4	40 (3.62)
TX	3 (0.27)
N classification	
NA	2 (0.18)
N0	516 (46.74)
N1	367 (33.24)
N2	120 (10.87)
N3	79 (7.16)
NX	20 (1.81)
M classification	
NA	2 (0.18)
M0	917 (83.06)
M1	22 (1.99)
MX	163 (14.76)
TNM stage	
NA	10 (0.91)
I	182 (16.49)
II	626 (56.7)
III	252 (22.83)
IV	20 (1.81)
X	14 (1.27)
Margin status	
NA	72 (6.52)
Close	31 (2.81)
Negative	922 (83.51)
Positive	79 (7.16)
Vital status	
NA	2 (0.18)
Deceased	155 (14.04)
Living	947 (85.78)
Radiation therapy	
NA	102 (9.24)
No	445 (40.31)
Yes	557 (50.45)
Neoadjuvant treatment	
NA	3 (0.27)
No	1088 (98.55)
Yes	13 (1.18)
Targeted molecular therapy	
NA	525 (47.55)
No	46 (4.17)
Yes	533 (48.28)
Sample type	
Metastatic	7 (0.63)
Primary tumor	1097 (99.37)
OS	
NA	17 (1.54)
0	933 (84.51)
1	154 (13.95)
RFS	
NA	192 (17.39)
0	816 (73.91)
1	96 (8.70)
ZNF385B	
High	804 (72.83)
Low	300 (27.17)

ER: estrogen receptor; PR: progesterone receptor; HER-2: human epidermal growth factor-2; T: tumor; M: metastasis; N: node; OS: overall survival; RFS: relapse-free survival; NA: not available.

**Table 2:** Correlation between clinicopathological characteristics and ZNF385B expression in breast cancer patients' samples.

Parameters	Variable	*N*	ZNF385B expression		*χ* ^2^	*p* value
			High (*N*%)	Low (*N*%)		
Age	<60	589	436 (54.3)	153 (51.17)	0.856	0.355
	≥60	513	367 (45.7)	146 (48.83)		

Gender	Female	1090	795 (99)	295 (98.66)	0.025	0.873
	Male	12	8 (1)	4 (1.34)		

Histological type	Infiltrating ductal carcinoma	790	551 (68.7)	239 (79.93)	22.841	<0.001
	Infiltrating lobular carcinoma	204	176 (21.95)	28 (9.36)		
	Other	107	75 (9.35)	32 (10.7)		

Molecular subtype	Basal	142	76 (12.1)	66 (29.86)	61.651	<0.001
	HER-2	67	44 (7.01)	23 (10.41)		
	LumA	422	355 (56.53)	67 (30.32)		
	LumB	194	132 (21.02)	62 (28.05)		
	Normal	24	21 (3.34)	3 (1.36)		

ER	Indeterminate	2	1 (0.13)	1 (0.35)	43.781	<0.001^b^
	Negative	239	134 (17.4)	105 (36.97)		
	Positive	813	635 (82.47)	178 (62.68)		

PR	Indeterminate	4	3 (0.39)	1 (0.35)	71.900	<0.001^b^
	Negative	345	194 (25.19)	151 (53.36)		
	Positive	704	573 (74.42)	131 (46.29)		

HER-2	Equivocal	180	127 (18.84)	53 (21.46)	3.960	0.266
	Indeterminate	12	7 (1.04)	5 (2.02)		
	Negative	565	425 (63.06)	140 (56.68)		
	Positive	164	115 (17.06)	49 (19.84)		

Menopause status	Inde	34	21 (2.86)	13 (4.71)	3.811	0.283
	Peri	40	32 (4.35)	8 (2.9)		
	Post	706	509 (69.25)	197 (71.38)		
	Pre	231	173 (23.54)	58 (21.01)		

T classification	T1	281	220 (27.4)	61 (20.4)	8.685	0.069
	T2	640	455 (56.66)	185 (61.87)		
	T3	138	102 (12.7)	36 (12.04)		
	T4	40	24 (2.99)	16 (5.35)		
	TX	3	2 (0.25)	1 (0.33)		

N classification	N0	516	374 (46.58)	142 (47.49)	6.606	0.158
	N1	367	276 (34.37)	91 (30.43)		
	N2	120	85 (10.59)	35 (11.71)		
	N3	79	58 (7.22)	21 (7.02)		
	NX	20	10 (1.25)	10 (3.34)		

M classification	M0	917	669 (83.31)	248 (82.94)	4.062	0.131
	M1	22	12 (1.49)	10 (3.34)		
	MX	163	122 (15.19)	41 (13.71)		

TNM stage	I	182	145 (18.19)	37 (12.46)	10.100	0.039
	II	626	448 (56.21)	178 (59.93)		
	III	252	184 (23.09)	68 (22.9)		
	IV	20	10 (1.25)	10 (3.37)		
	X	14	10 (1.25)	4 (1.35)		

Margin status	Close	31	24 (3.2)	7 (2.47)	0.596	0.742
	Negative	922	666 (88.92)	256 (90.46)		
	Positive	79	59 (7.88)	20 (7.07)		

Vital status	Deceased	155	91 (11.33)	64 (21.4)	18.287	<0.001
	Living	947	712 (88.67)	235 (78.6)		

Radiation therapy	No	445	319 (43.58)	126 (46.67)	0.762	0.383
	Yes	557	413 (56.42)	144 (53.33)		

Neoadjuvant treatment	No	1088	795 (99)	293 (98.32)	0.380	0.538
	Yes	13	8 (1)	5 (1.68)		

Targeted molecular therapy	No	46	34 (7.96)	12 (7.89)	0.001	0.979
	Yes	533	393 (92.04)	140 (92.11)		

Sample type	Metastatic	7	6 (0.75)	1 (0.33)	0.118	0.732
	Primary tumor	1097	798 (99.25)	299 (99.67)		

OS	0	933	702 (88.52)	231 (78.57)	17.473	<0.001
	1	154	91 (11.48)	63 (21.43)		

RFS	0	816	617 (91.54)	199 (83.61)	11.743	<0.001
	1	96	57 (8.46)	39 (16.39)		

ER: estrogen receptor; PR: progesterone receptor; HER-2: human epidermal growth factor-2; T: tumor; M: metastasis; N: node; OS: overall survival; RFS: relapse-free survival. ^b^Fisher's exact test.

**Table 3:** Univariate and multivariate Cox regression analyses of various prognostic parameters of overall survival in patients with breast cancer.

Parameters	Univariate analysis		Multivariate analysis	
	HR (95% CI)	*p* value	HR (95% CI)	*p* value
Age (years) (≥60 vs.<60)	1.912 (1.390-2.631)	<0.001	2.249 (1.219-4.148)	0.009
Menopause status				
Peri vs. pre	0.369 (0.049-2.761)	0.332	0.822 (0.108-6.284)	0.850
Post vs. pre	2.105 (1.281-3.459)	0.003	1.228 (0.585-2.581)	0.587
T classification				
T2 vs. T1	1.331 (0.887-1.998)	0.167	1.198 (0.632-2.271)	0.580
T3 vs. T1	1.578 (0.936-2.658)	0.087	1.484 (0.605-3.643)	0.389
T4 vs. T1	3.967 (2.138-7.362)	<0.001	1.721 (0.534-5.547)	0.363
N classification				
N1 vs. N0	1.874 (1.273-2.758)	0.001	1.231 (0.638-2.372)	0.535
N2 vs. N0	2.719 (1.627-4.545)	<0.001	3.195 (0.974-10.478)	0.055
N3 vs. N0	4.081 (2.256-7.381)	<0.001	4.463 (1.401-14.222)	0.011
M classification (M1 vs. M0)	4.784 (2.858-8.007)	<0.001	3.504 (1.393-8.815)	0.008
TNM stage (III-IV vs. I-II)	2.605 (1.869-3.630)	<0.001	1.187 (0.415-3.391)	0.749
Margin status				
Close vs. negative	1.876 (0.818-4.303)	0.137	2.740 (1.028-7.306)	0.044
Positive vs. negative	1.951 (1.186-3.212)	0.009	0.647 (0.296-1.417)	0.277
ZNF385B (low vs. high)	2.021 (1.465-2.788)	<0.001	3.040 (1.894-4.877)	<0.001

HR: hazard ratio; CI: confidence interval; M: metastasis; N: node; T: tumor.

**Table 4:** Univariate and multivariate Cox regression analyses of various prognostic parameters of relapse-free survival in patients with breast cancer.

Parameters	Univariate analysis		Multivariate analysis	
	HR (95% CI)	*p* value	HR (95% CI)	*p* value
ER (negative vs. positive)	1.643 (1.062-2.542)	0.026	1.340 (0.611-2.937)	0.465
PR (negative vs. positive)	1.641 (1.088-2.477)	0.018	1.402 (0.657-2.994)	0.383
T classification				
T2 vs. T1	1.635 (0.944-2.833)	0.079	1.235 (0.631-2.417)	0.537
T3 vs. T1	2.524 (1.321-4.824)	0.005	1.267 (0.487-3.299)	0.628
T4 vs. T1	6.72 (2.885-15.656)	<0.001	2.013 (0.553-7.321)	0.288
N classification				
N1 vs. N0	2.255 (1.357-3.748)	0.002	1.560 (0.822-2.958)	0.173
N2 vs. N0	2.642 (1.326-5.264)	0.006	2.741 (0.749-10.035)	0.128
N3 vs. N0	6.54 (3.415-12.524)	<0.001	7.106 (1.998-25.278)	0.002
M classification (M1 vs. M0)	4.024 (1.742-9.292)	0.001	1.294 (0.442-3.784)	0.638
TNM stage (III-IV vs. I-II)	3.190 (2.106-4.831)	<0.001	1.147 (0.375-3.508)	0.809
Margin status				
Close vs. negative	2.228 (0.966-5.141)	0.060	3.229 (1.319-7.905)	0.010
Positive vs. negative	2.453 (1.441-4.177)	0.001	1.738 (0.836-3.611)	0.139
ZNF385B (low vs. high)	2.227 (1.480-3.349)	<0.001	2.609 (1.531-4.449)	<0.001

CI: confidence interval; ER: estrogen receptor; HR: hazard ratio; M: metastasis; N: node; PR: progesterone receptor; T: tumor.

## Data Availability

The data used to support the findings of this study are included within the article.

## Abbreviations

AUC: Area under the curve
BC: Breast cancer
GEO: Gene Expression Omnibus
HR: Hazard ratio
IHC: Immunohistochemistry
OS: Overall survival
RFS: Relapse-free survival
ROC: Receiver operating characteristic
TCGA: The Cancer Genome Atlas.

## Supplementary Materials

Supplementary 1 Supplementary Figure 1: pan-cancer analysis of ZNF385B mRNA expression based on Oncomine database and “ESurv” tool.

Supplementary 2 Supplementary Figure 2: the ROC curves determining the optimal cut-off values of high and low ZNF385B expression in TCGA and GEO databases.
