# Supplementary figures and images for: Downregulated mRNA Expression of ZNF385B Is an Independent Predictor of Breast Cancer
Source: Int J Genomics. 2021 Feb 3;2021:4301802. doi: 10.1155/2021/4301802 (PMC7876827; doi:10.1155/2021/4301802)

## Slide 1
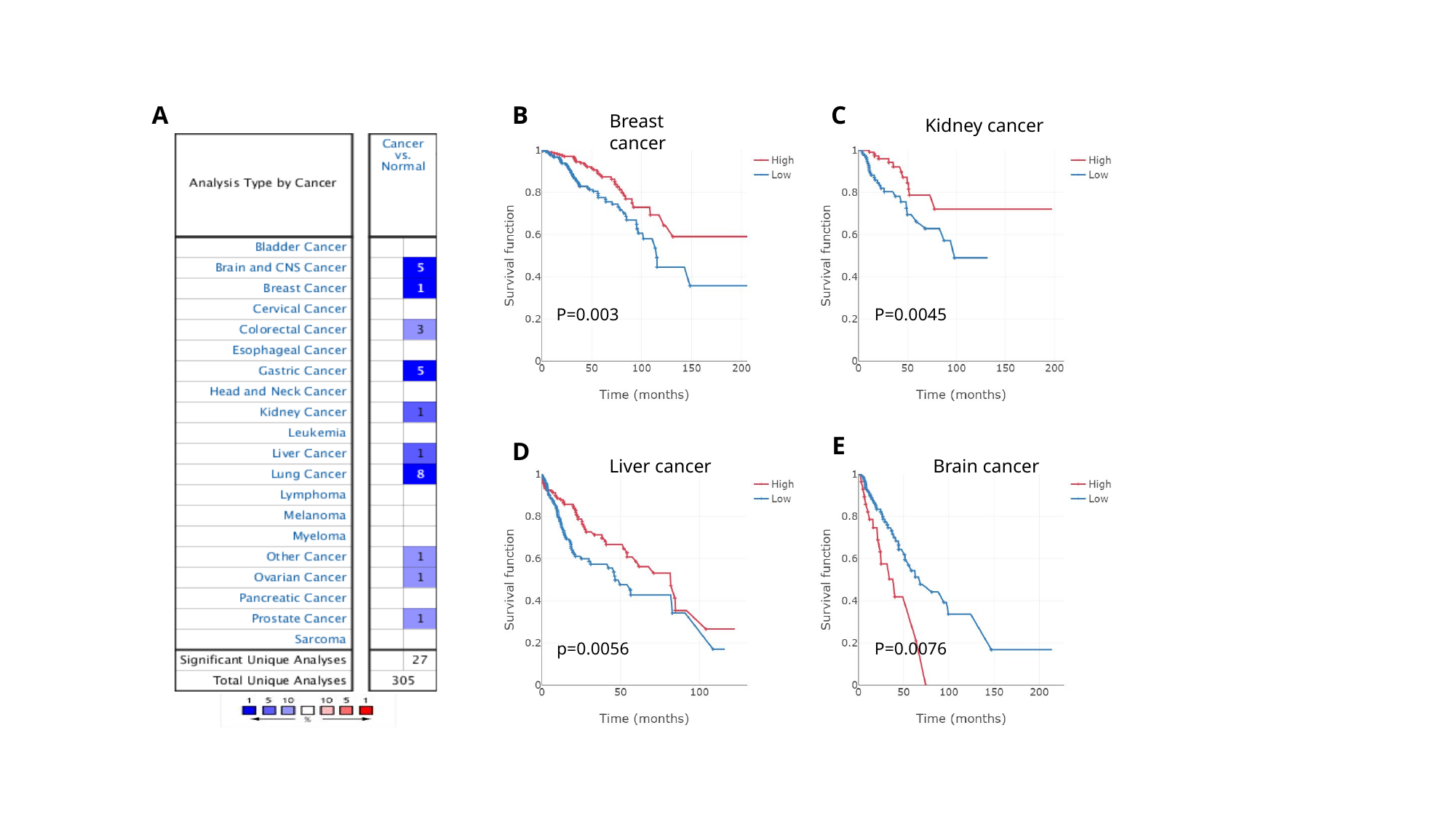

A
B
Breast cancer
P=0.003
C
Kidney cancer
P=0.0045
D
Liver cancer
p=0.0056
E
Brain cancer
P=0.0076

Supplement: Supplementary 1 — Supplementary Figure 1: pan-cancer analysis of ZNF385B mRNA expression based on Oncomine database and “ESurv” tool. [file 4301802.f1.pptx]

## Slide 1
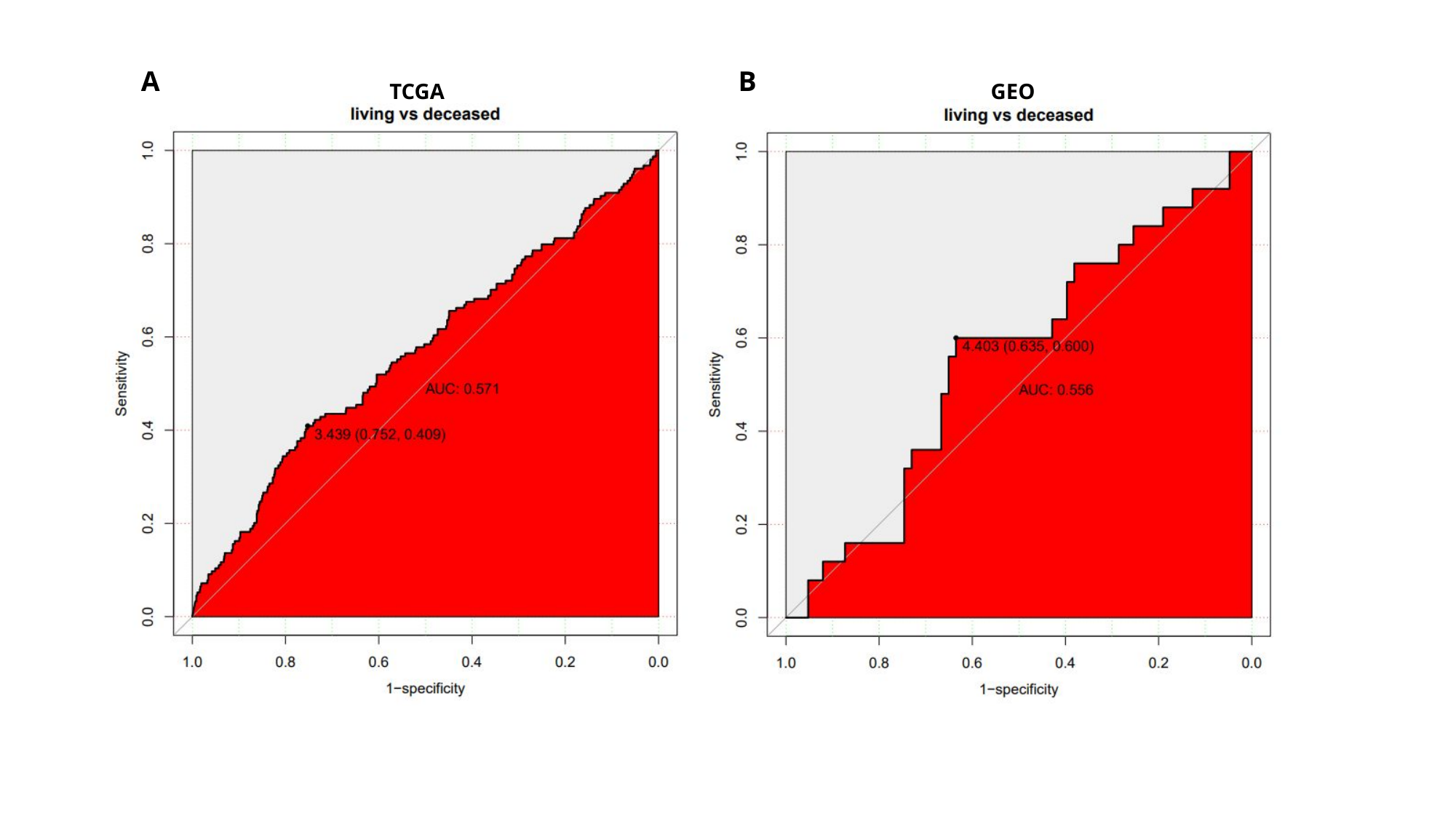

A
B
TCGA
GEO

Supplement: Supplementary 2 — Supplementary Figure 2: the ROC curves determining the optimal cut-off values of high and low ZNF385B expression in TCGA and GEO databases. [file 4301802.f2.pptx]
